# A case of non‐severe COVID‐19 complicated by pulmonary embolism

**DOI:** 10.1002/rcr2.622

**Published:** 2020-07-15

**Authors:** Yuto Akiyama, Kohei Horiuchi, Yasushi Kondo, Hiroki Kabata, Makoto Ishii, Koichi Fukunaga

**Affiliations:** ^1^ Division of Pulmonary Medicine, Department of Medicine Keio University School of Medicine Shinjuku Tokyo Japan; ^2^ Division of Rheumatology, Department of Medicine Keio University School of Medicine Shinjuku Tokyo Japan

**Keywords:** COVID‐19, d‐dimer, Padua prediction score, pulmonary embolism, SARS‐CoV‐2

## Abstract

Novel coronavirus disease 2019 (COVID‐19) caused by severe acute respiratory syndrome coronavirus 2 is rapidly spreading worldwide. A typical clinical manifestation of COVID‐19 is pneumonia, which can progress to acute respiratory distress syndrome and respiratory failure. Recent studies have reported that COVID‐19 is often accompanied by coagulopathy, and a significant number of patients with severe or critical COVID‐19 develop concomitant thrombosis, including pulmonary embolism (PE). However, there are limited reports of the incidence of PE in non‐severe COVID‐19 patients. Here, we report a case of non‐severe COVID‐19 complicated by PE, which indicates that the possibility of PE should consistently be considered, even in non‐severe cases of COVID‐19 without any risk of thrombosis.

## Graphical abstract

Here, we report a case of non‐severe coronavirus disease 2019 (COVID‐19) complicated by pulmonary embolism (PE), which indicates that the possibility of PE should consistently be considered, even in non‐severe cases of COVID‐19 without any risk of thrombosis.

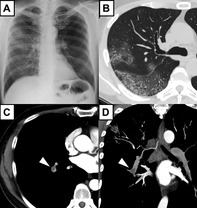

## Introduction

There have been several reports of the occurrence of coagulopathy in patients with novel coronavirus disease 2019 (COVID‐19). It has been proposed that vascular endotheliitis caused by either an activated immune reaction or direct infection of the vascular endothelium with severe acute respiratory syndrome coronavirus 2 (SARS‐CoV‐2) may cause coagulopathy. However, the pathophysiology of COVID‐19‐associated coagulopathy is not well understood. The occurrence of pulmonary embolism (PE) has been reported in COVID‐19 patients, mainly in Europe and the USA. The incidence of PE is high in severe or critical COVID‐19 cases requiring intensive care unit (ICU) admission [1]. However, there have been few reports of PE associated with COVID‐19 in Japan, and PE is a rare complication of non‐severe COVID‐19. Therefore, the incidence of PE in patients with non‐severe COVID‐19 and measures to best prevent coagulopathy have not been elucidated. Here, we report a case of non‐severe COVID‐19 without any risk of thrombosis that was diagnosed as PE based on prolonged serum d‐dimer elevation.

## Case Report

A 56‐year‐old man with no comorbidities developed a 39°C fever nine days before being admitted to our hospital. Five days later, he visited a nearby clinic due to persistent fever, and chest X‐ray revealed pneumonia. A polymerase chain reaction (PCR)‐based test for SARS‐CoV‐2 was performed, and he was diagnosed with COVID‐19 based on his positive PCR result. The patient was then referred to our hospital.

**Figure 1:**
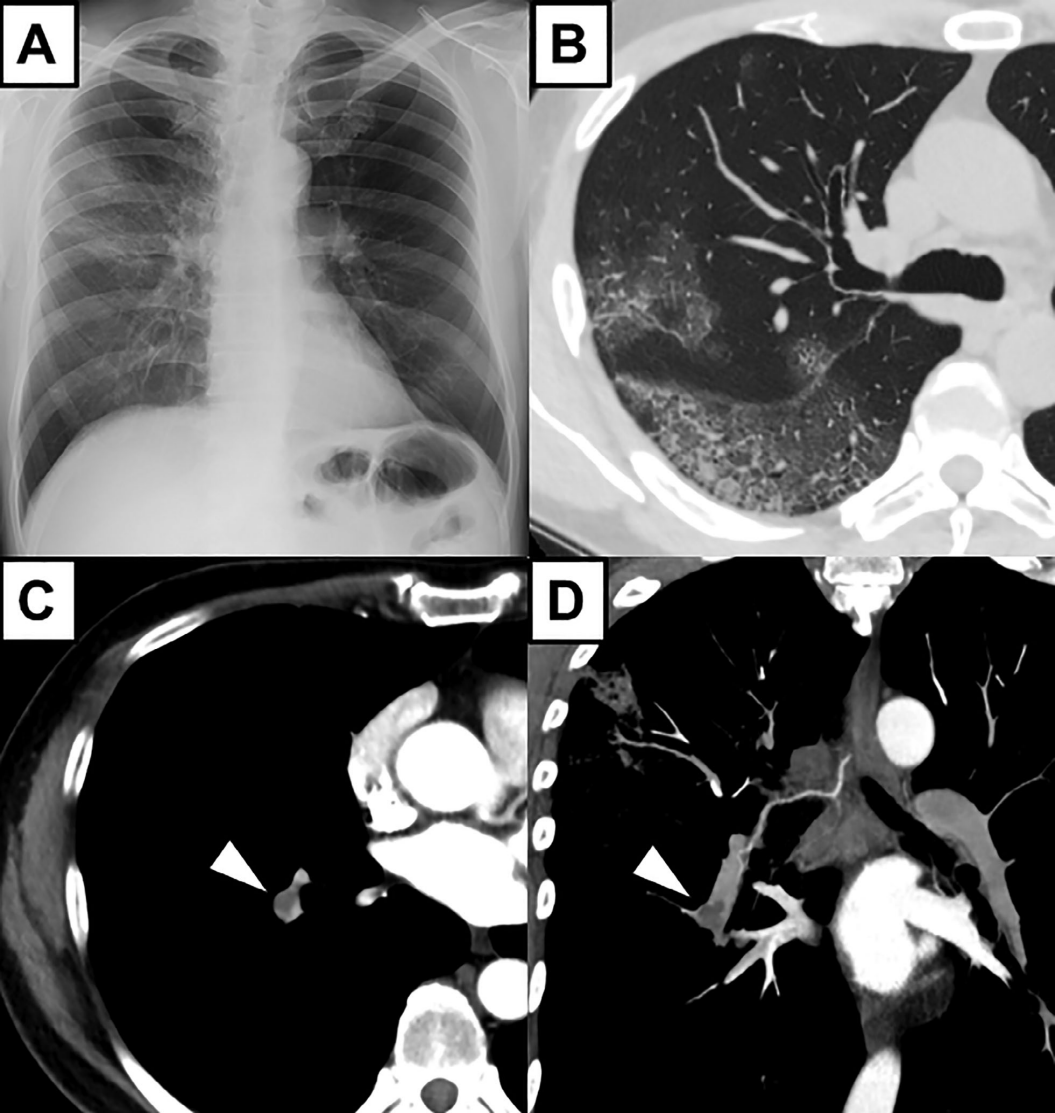
(A) Chest X‐ray on admission. Ground‐glass opacity (GGO) was observed in the right lower and middle lung field. (B) Chest computed tomography (CT) on admission showed GGO in the upper lobe and S6 region of the right lung. (C, D) Chest contrast CT on day 5 showed poor contrast enhancement in the right pulmonary artery, suggesting pulmonary embolism (PE) (white arrowheads).

On admission, he presented with a pulse of 78 bpm, body temperature of 37.8°C, blood pressure of 128/90 mmHg, and 97% oxygen saturation when breathing air. At admission, laboratory data were as follows: lactate dehydrogenase 267 U/L, aspartate aminotransferase 35 U/L, alanine aminotransferase 47 U/L, C‐reactive protein (CRP) 7.91 mg/dL, activated partial thromboplastin time 23.8 sec, prothrombin time‐international normalized ratio 1.19, and d‐dimer 1.0 μg/mL. No lymphopenia was noted, and the patient's electrocardiogram was normal. His chest X‐ray revealed ground‐glass opacity (GGO) of the right lower and middle lung field (Fig. 1A). His chest computed tomography (CT) showed GGO in the right upper lobe and S6 region of the lung (Fig. 1B). We initiated favipiravir for COVID‐19. As the Padua prediction score for the patient was 1, venous thromboembolism risk was considered low and no therapeutic thromboprophylaxis treatment was administered [2].

**Figure 2:**
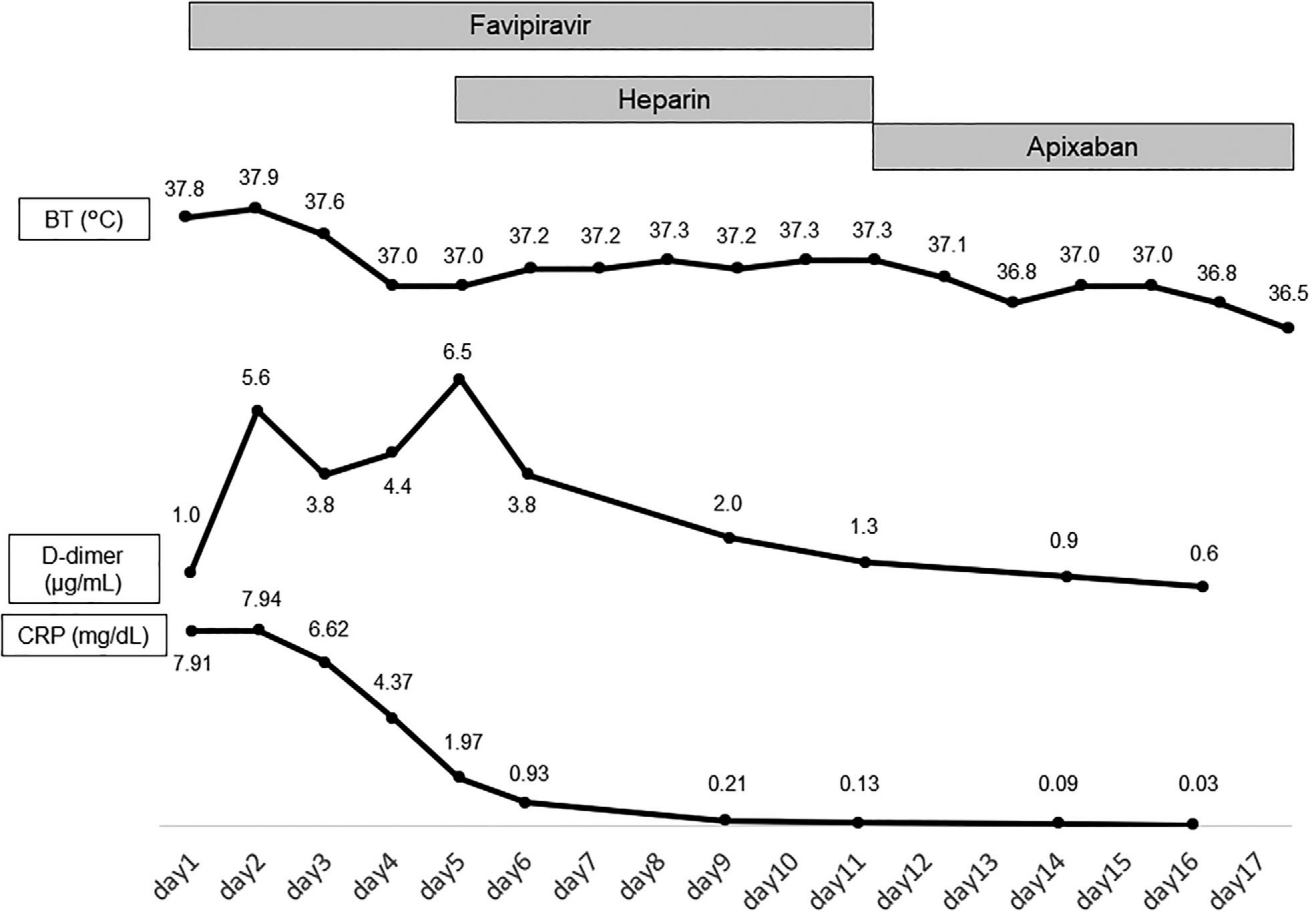
Clinical course of the case. On admission, favipiravir was started against coronavirus disease 2019 (COVID‐19). On day 5, his fever and inflammatory markers, including C‐reactive protein (CRP), improved significantly. However, d‐dimer was elevated to 6.5 μg/mL. We diagnosed pulmonary embolism (PE) by contrast computed tomography (CT) and started the administration of heparin. After anticoagulation therapy, d‐dimer was gradually decreased. We switched from heparin to apixaban on day 11, and he was discharged on day 17.

On day 2 post‐hospital admission, laboratory findings included 7.94 mg/L CRP, 686 ng/mL ferritin, and 5.6 μg/mL d‐dimer. d‐dimer levels were only slightly elevated, there were no changes with his haemodynamics or respiratory status (oxygen saturation: 94% on room air), and the patient experienced neither oedema nor gripping pain in his lower extremities. Three days post‐hospital admission, his fever and CRP levels gradually improved, and five days post‐admission, his fever had almost resolved, inflammatory markers, such as CRP (1.97 mg/L) and ferritin (366 ng/mL), were significantly improved. In addition, his PCR test for SARS‐CoV‐2 was negative. However, d‐dimer levels remained elevated (6.5 μg/mL) and deviated from the other clinical and laboratory findings. Although he had no symptoms including dyspnoea even when he walked and respiratory status was not changed (oxygen saturation: 97% on room air), we performed contrast CT in order to identify PE and deep vein thrombosis (DVT). Contrast CT revealed PE of the right pulmonary artery (Fig. 1C, D), while no DVT or other thrombosis was detected. We initiated continuous heparin infusion to treat PE. Screening tests for autoimmune disease and congenital coagulation abnormalities, such as activated protein C, activated protein S, antinuclear antibody, anti‐double stranded DNA, immunoglobulin G, anti‐cardiolipin‐β2‐glycoprotein I complex antibody, and lupus anticoagulant were all negative. On day 11 post‐admission, we replaced heparin with apixaban. d‐dimer levels gradually decreased by day 11 post‐admission (1.3 μg/mL), and the patient was discharged after being hospitalized for 17 days (Fig. 2).

## Discussion

PE is an important complication of COVID‐19, particularly in severe or critical COVID‐19 patients [1]. However, there have been few reports of PE complicated in patients with non‐severe COVID‐19.

In this case, we suspected PE based on increased d‐dimer levels. Although d‐dimer levels are commonly used to screen for PE, it has been reported that d‐dimers are elevated in 46.4% of patients with COVID‐19 [3]. Therefore, the University of Michigan Health System has indicated that increased d‐dimer levels are not useful for predicting COVID‐19‐associated PE [4]. Alternatively, different study reported that d‐dimers could be used to predict PE in COVID‐19 patients with 100% and 67% sensitivity and specificity, respectively, when a d‐dimer cut‐off level of 2.66 μg/mL was used [5]. Here, D‐dimer levels peaked at 5.6 μg/mL, and PE was suspected as a result of prolonged elevation of d‐dimer levels, which persisted despite the improvement of other inflammatory parameters. Thus, our assessment depended on both the concentration of d‐dimer observed and the clinical course of COVID‐19.

There are currently no established criteria that specify the use of prophylactic anticoagulant therapy in COVID‐19 patients. Further clinical trials will be needed to determine whether all COVID‐19 patients should be treated with an anticoagulant. At this time, it is important to proactively explore the possibility of PE based on clinical presentation and d‐dimer levels, even in non‐severe COVID‐19 cases.

### Disclosure Statements

Appropriate written informed consent was obtained for publication of this case report and accompanying images.

At the time this report was accepted for publication, the authors declared that the patient in this report had not been included in any previously published report on COVID‐19 that they had authored.

## References

[1] Lodigiani C , Iapichino G , Carenzo L , et al. 2020 Venous and arterial thromboembolic complications in COVID‐19 patients admitted to an academic hospital in Milan, Italy. Thromb. Res. 191:9–14. 32353746 10.1016/j.thromres.2020.04.024 PMC7177070

[2] Barbar S , Noventa F , Rossetto V , et al. 2010 A risk assessment model for the identification of hospitalized medical patients at risk for venous thromboembolism: the Padua Prediction Score. J. Thromb. Haemost. 8(11):2450–2457. 20738765 10.1111/j.1538-7836.2010.04044.x

[3] Guan WJ , Ni ZY , Liang WH , et al. 2020 Clinical characteristics of coronavirus disease 2019 in China. N. Engl. J. Med. 382(18):1708–1720. 32109013 10.1056/NEJMoa2002032 PMC7092819

[4] Obi AT , Barnees GD , Wakefield TW , et al. 2020 Practical diagnosis and treatment of suspected venous thromboembolism during COVID‐19 pandemic. J. Vasc. Surg. Venous Lymphat. Disord. 8:526–534. 10.1016/j.jvsv.2020.04.009. 32305585 PMC7162794

[5] Leonard‐Lorant I , Delabranche X , Severac F , et al. 2020 Acute pulmonary embolism in COVID‐19 patients on CT angiography and relationship to D‐dimer levels. Radiology:201561 [Epub ahead of print]. 10.1148/radiol.2020201561. 32324102 PMC7233397

